# Patterns of Inter-Chromosomal Gene Conversion on the Male-Specific Region of the Human Y Chromosome

**DOI:** 10.3389/fgene.2017.00054

**Published:** 2017-05-03

**Authors:** Beniamino Trombetta, Eugenia D’Atanasio, Fulvio Cruciani

**Affiliations:** ^1^Dipartimento di Biologia e Biotecnologie “Charles Darwin”, Sapienza Università di RomaRome, Italy; ^2^Istituto di Biologia e Patologia Molecolari, Consiglio Nazionale delle Ricerche (CNR),Rome, Italy

**Keywords:** gene conversion, Y chromosome, sex chromosome evolution, nucleotide diversity, cluster of SNPs

## Abstract

The male-specific region of the human Y chromosome (MSY) is characterized by the lack of meiotic recombination and it has long been considered an evolutionary independent region of the human genome. In recent years, however, the idea that human MSY did not have an independent evolutionary history begun to emerge with the discovery that inter-chromosomal gene conversion (ICGC) can modulate the genetic diversity of some portions of this genomic region. Despite the study of the dynamics of this molecular mechanism in humans is still in its infancy, some peculiar features and consequences of it can be summarized. The main effect of ICGC is to increase the allelic diversity of MSY by generating a significant excess of clustered single nucleotide polymorphisms (SNPs) (defined as groups of two or more SNPs occurring in close proximity and on the same branch of the Y phylogeny). On the human MSY, 13 inter-chromosomal gene conversion hotspots (GCHs) have been identified so far, involving donor sequences mainly from the X-chromosome and, to a lesser extent, from autosomes. Most of the GCHs are evolutionary conserved and overlap with regions involved in aberrant X–Y crossing-over. This review mainly focuses on the dynamics and the current knowledge concerning the recombinational landscape of the human MSY in the form of ICGC, on how this molecular mechanism may influence the evolution of the MSY, and on how it could affect the information enclosed within a genomic region which, until recently, appeared to be an evolutionary independent unit.

## Introduction

Human sex chromosomes (the X and Y chromosome) originated from a single pair of ancestral recombining autosomes (proto-sex chromosomes) that began to differentiate between 160 and 190 million years ago (Mya), after the split of monotremes from Theria (Luo et al., 2011; Katsura et al., 2012). This differentiation started with the emergence of a male-determining gene on the proto-Y chromosome and the progressive accumulation of genes with male-specific functions on it. Notably, to maintain new male-specific genes on the proto-Y, natural selection favored the suppression of meiotic recombination between proto-sex chromosomes (Ellegren, 2011; Bachtrog, 2013; Hughes and Page, 2015). As a consequence, the evolution of the Y chromosome has been characterized by a rapid structural decay and the loss of most of its ancestral genes (Skaletsky et al., 2003; Hughes et al., 2005, 2010, 2012; Bachtrog, 2008; Li et al., 2013; Hughes and Page, 2015).

The suppression of meiotic recombination in the heterogametic sex occurred in at least five distinct steps (Lahn and Page, 1999; Ross et al., 2005; Pandey et al., 2013). These events generated five discrete clusters (termed “evolutionary strata”—from 1 to 5) with specific X–Y nucleotide divergence depending on the time of recombination arrest (Skaletsky et al., 2003; Ross et al., 2005). The oldest stratum was generated in the stem lineage of Theria (Bininda-Emonds et al., 2007; Veyrunes et al., 2008; Katsura and Satta, 2012), whereas the youngest stratum (stratum 5), which retains the highest X–Y sequence similarity (∼95%), originated only 30 Mya (Ross et al., 2005; Hughes et al., 2012).

The telomeric portions of X and Y chromosomes (pseudoautosomal regions, PARs) share 100% sequence identity and recombine each other during male gametogenesis. On the Y chromosome, PARs mark the boundaries of a male-specific region of the human Y chromosome (MSY) comprising 95% of the entire chromosome. The MSY is a mosaic of three classes of sequences: X-transposed, X-degenerate, and ampliconic (Skaletsky et al., 2003). The X-transposed regions originated from an X-to-Y transposition 4.7 Mya (Ross et al., 2005). The X-degenerate sequences are remnants of the proto-sex chromosomes and contain all the evolutionary strata. The ampliconic sequences are mainly composed of eight palindromic structures (termed P1–P8), each of which consists of two highly similar inverted paralogs (or “arms”) separated by a non-duplicated spacer sequence. Palindromic sequences show an arm-to-arm nucleotide identity >99.9%, due to frequent intra-chromosomal gene conversion events (i.e., the non-reciprocal transfer of genetic information between two homologous sequences; Rozen et al., 2003; Hallast et al., 2013).

With the exclusion of ancient episodic X–Y gene conversion events (Pecon Slattery et al., 2000; Iwase et al., 2010), which occurred during sex chromosome evolution, and very rare illegitimate crossing over events which have generated chromosomal aberrations (Vollrath et al., 1992; Schiebel et al., 1997), the MSY has long been considered an evolutionary independent region of the human genome.

This view has been recently dismissed by the discovery that the sequence landscape of the human MSY can be modulated by inter-chromosomal gene conversion (ICGC) which may occur in narrow portions of the X-degenerate region called gene conversion hotspots (GCHs) (Rosser et al., 2009; Cruciani et al., 2010; Trombetta et al., 2010, 2014, 2016; Niederstätter et al., 2013). All these studies suggest that the GCHs are not fully Y-linked regions and that the sequence of the MSY is patchy, with regions that can exchange variants between X (or autosomes) and Y chromosome by gene conversion, while other regions remain genetically isolated. Gene conversion between sex chromosomes is potentially bidirectional (both X-to-Y and Y-to-X), although Y-to-X has not been extensively studied (Trombetta et al., 2014) due to the confounding factor introduced by crossing-over between X homologs in female meiosis.

This review mainly focuses on current knowledge concerning the recombinational landscape of the human MSY in the form of ICGC, on how this molecular mechanism may influence the evolution of the MSY, and on how it could affect the information enclosed within a genomic region which, until recently, appeared to be independently transmitted.

## Inter-Chromosomal Gene Conversion on Human Msy

Gene conversion is a particular kind of recombination, which (differently from crossing over) consists in the unidirectional transfer of genetic information from a “donor” sequence to a highly similar “acceptor” (Chen et al., 2007). The genetic transfer can occur between allelic sequences or between highly similar (identity >80%) non-allelic (ectopic) regions located on the same or on different chromosomes (Chen et al., 2007). Gene conversion always initiates with a DNA double-strand break (DSB) in the acceptor sequence and it is the main molecular mechanism used to repair DSBs within the human genome (Chen et al., 2007). During this process, the broken region uses the intact paralogous strand as a template to repair itself and its main effect is that the acceptor sequence becomes identical to the donor, which remains unchanged (Szostak et al., 1983).

Two forms of gene conversion are known to involve the MSY. The most common is Y–Y gene conversion mainly occurring between the palindrome arms of the ampliconic region (Rozen et al., 2003). It has been proposed that this mechanism could have evolved to counteract the debilitating consequences of the absence of recombination on some important genes of the Y chromosome (Charlesworth, 2003; Rozen et al., 2003; Betrán et al., 2012; Hallast et al., 2013; Trombetta and Cruciani, 2017). The other form is ICGC, where either X chromosome or autosomal sequences replace paralogous regions on the Y chromosome (Rosser et al., 2009; Cruciani et al., 2010; Trombetta et al., 2010, 2014, 2016).

ICGC on the MSY was first identified in three narrow X-to-Y GCHs. One hotspot is situated within the *VCY* genes, in the P8 palindrome, whereas the other two hotspots are located in evolutionary stratum 5 of the X-degenerate region, in the *ARSDP* pseudogene (Trombetta et al., 2010; Niederstätter et al., 2013) and near the 5′-end of the *PRKY* gene (Rosser et al., 2009; Cruciani et al., 2010). Successively, other six GCHs have been found to be active on human MSY, one of which is characterized by autosome-to-Y gene conversion (Trombetta et al., 2014, 2016). Moreover, using a phylogenetic approach, X–Y gene conversion has been hypothesized for at least four other narrow regions of stratum 5 (**Table 1**). However, it was not possible to determine whether these four regions were still active GCHs or simply cold spots that had experienced ancient X–Y gene conversion events (Trombetta et al., 2014).

**Table 1:** Inter-chromosomal gene conversion hotspots on the human MSY.

GCH	Position on the Y chromosome^a^	Length	Donor-to-acceptor	Active or historical GCH	GCH in chimp	Number of donors	Unfiltered SNPs^b^	Recurring SNPs^b^	Filtered SNPs^b^	Reference
HSA	7096711–7097641	931	X-to-Y	Active	Yes	Single donor	4	0	0	Rosser et al., 2009
CER3	7163099–7163862	776	X-to-Y	Historical	Yes	Single donor	2	0	2	Trombetta et al., 2014
CER6	7169931–7170442	493	X-to-Y	Active	No	Single donor	1	0	0	Trombetta et al., 2014
CER7	7171778–7173059	1283	X-to-Y	Active	Yes	Single donor	5	0	2	Trombetta et al., 2014
CER9	7234931–7235419	489	X-to-Y	Historical	Yes	Single donor	3	0	0	Trombetta et al., 2014
CER12	7319098–7319526	429	X-to-Y	Historical	Yes	Single donor	3	0	3	Trombetta et al., 2014
CER15	14051551–14051623	73	X-to-Y	Active	Yes	Single donor	0	0	0	Trombetta et al., 2014
CER17	14483248–14484067	884	X-to-Y	Active	Yes	Single donor	4	0	3	Trombetta et al., 2014
CER18	14484255–14484908	655	X-to-Y	Active	No	Single donor	6	0	3	Trombetta et al., 2014
CER19	14490130–14490661	535	X-to-Y	Historical	Yes	Single donor	2	1	0	Trombetta et al., 2014
LTR2	2865434–2866983	1549	X-to-Y/autosome-to-Y	Active	–	Multiple donors	39	6	13	Trombetta et al., 2016
ARSDP	14491586–14491933	348	X-to-Y	Active	–	Single donor	5	1	1	Trombetta et al., 2010
VCY	16097652–16098392	740	X-to-Y	Active	Yes	Multiple donors	0	0	0	Trombetta et al., 2010
	16168098 –16168838									

*^a^Genomic position according to GChR37/hg19.*

*^b^Data from Karmin et al. (2015).*

Gene conversion is known to increase sequence similarity between the interacting paralogs (Hurles and Jobling, 2001; Korunes and Noor, 2017). The dramatic impact of this sequence homogenization can be observed in the *VCY* hotspots within the stratum 4 (Trombetta et al., 2010). The average X–Y nucleotide identity in stratum 4 is about 87% (due to the stop in recombination which occurred about 40 Mya; Skaletsky et al., 2003), whereas the sequence similarity for *VCX/VCY* genes is above 98% (Ross et al., 2005).

As well as increasing sequence identity between paralogs, ICGC can also be highly effective in determining a rise in the single nucleotide polymorphism (SNP) content of GCHs (Nielsen et al., 2003; Trombetta et al., 2010, 2016). Indeed, all the active GCHs identified so far on the human MSY show a higher allelic diversity when compared with their surrounding regions and with the average diversity of the entire chromosome (Trombetta et al., 2010, 2014, 2016).

Different hotspots show very different values of nucleotide diversity (Cruciani et al., 2010; Trombetta et al., 2010, 2014, 2016). Interestingly, the level of allelic diversity in an acceptor sequence seems to be related not only to the rate of gene conversion, but also to the number of different donor sequences involved. The presence of more than one donor sequence is a continuous source of variants and it can greatly increase the allelic diversity of the GCHs within the MSY. This is because the donor sequences are free to mutate and accumulate PSVs (paralogous sequence variants) continuously so that a dynamic balance between mutation and gene conversion will never lead to a complete sequence identity among the interacting sequences. As a matter of fact, the highest MSY nucleotide diversity figures have been observed at the *VCY* and LTR2 hotspots, both involving multiple donor sequences (Trombetta et al., 2010, 2016). On the contrary, when only one donor is involved, gene conversion events can only decrease the diversity between donor and acceptor sequences. Consequently, a conversion-mutation dynamic equilibrium will be reached and the similarity between interacting sequences will increase up to a point in which conversion events will no longer influence the genetic diversity of GCHs, due to the lack of differences between donor and acceptor.

In the human genome, ICGC tract length is highly variable (Zangenberg et al., 1995; Papadakis and Patrinos, 1999; Chen et al., 2007). The gene conversion events which involve the MSY as an acceptor sequence are generally very short. If one considers only the X-to-Y gene conversion events, the minimum observed tract length ranges from 1 to 86 bp, whereas the maximum tract ranges from 9 to 163 bp, with a mean value of 47 bp (Cruciani et al., 2010; Trombetta et al., 2010, 2014, 2016; Niederstätter et al., 2013). These figures are in the lower range of ectopic gene-conversion-tract lengths for autosomal hotspots (Eikenboom et al., 1994; Hallast et al., 2005; Chen et al., 2007). The overlap of the tract lengths observed for autosomic and sex chromosome ectopic gene conversion may suggest a similar molecular mechanism regardless of the chromosomal context in which it occurs.

## Features of Msy Gene Conversion Hotspots

To date, 13 GCHs have been identified within the human MSY (**Table 1**), nine of which are still active whereas the remaining show signals of ancient episodes of gene conversion with no evidences of recent events (Rosser et al., 2009; Cruciani et al., 2010; Trombetta et al., 2010, 2014, 2016; Niederstätter et al., 2013).

**FIGURE 1:**
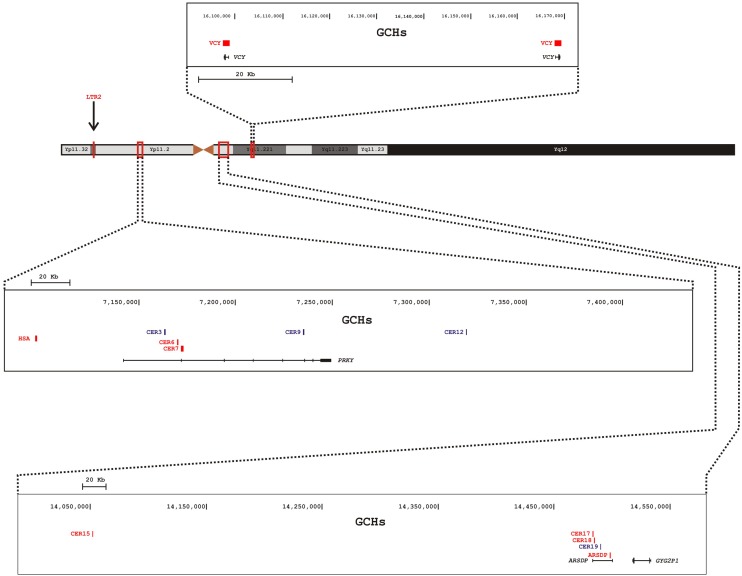
**Schematic representation of the Y chromosome and distribution of GCHs on the human MSY.** Nomenclature of GCHs is as in **Table 1**. Different boxes indicate different magnified regions of the MSY. In each box are reported (from the top): the genomic coordinates, the position of the GCHs, and the genes within the region. Active GCHs are colored in red, historical GCHs are colored in blue. The position of the LTR2 hotspot is indicated by an arrow.

These GCHs seem to be unevenly distributed on the Y chromosome (**Figure 1**). Out of the 13 hotspots, eight lie within two pseudogenes (*PRKY* and *ARSDP*) and one hotspot overlaps with the active *VCY* gene, while the remaining GCHs were found in intergenic regions. All these MSY GCHs have donor sequences on the X chromosome, whereas the LTR2 hotspot is also involved in autosome-to-Y gene conversion (**Table 1**; Trombetta et al., 2016).

Interestingly, there is a significant excess of exonic sequences covered by GCHs (Trombetta et al., 2014). This finding suggests that the possible functional differentiations between gametologous genes (which is a hallmark of sex chromosome evolution) can be erased by gene conversion. This could be an evolutionary cost that may be counterbalanced by the beneficial effects of gene-conversion-mediated repair of DSBs.

Most MSY ICGC hotspots identified so far share three major features: (1) they all have a size of about 1 kb (**Table 1**); (2) several of them are evolutionarily conserved, being also active in the chimpanzee lineage; and (3) almost all of the X-to-Y hotspots overlap with regions where X–Y crossing-over has been previously reported to be involved in sex reversal.

The existence of shared X–Y hotspots across two related species such as humans and chimpanzees may suggest that their origin predates human–chimp speciation and that they have been maintained active in both lineages (Iwase et al., 2010; Trombetta et al., 2010; Fawcett and Innan, 2013). This finding is at odds with the observation that allelic homologous recombination (AHR) hotspots minimally overlap between chimpanzee and human species (Ptak et al., 2004; Winckler et al., 2005; Auton et al., 2012; Fawcett and Innan, 2013), whereas it is in line with previous reports of human–chimp conserved intra-chromosomal non-AHR (NAHR) hotspots in the Y chromosome (Rozen et al., 2003; Bosch et al., 2004; Hurles et al., 2004; Lee et al., 2008; Perry et al., 2008; Trombetta and Cruciani, 2017). These observations suggest a longer evolutionary lifespan of NAHR hotspots compared with the AHR ones.

Gene conversion is a common outcome in recombination-mediated DSB repair processes. This molecular mechanism leads to the formation of a “Holliday junction” that can be resolved by either gene conversion or crossing-over (Hunter and Kleckner, 2001; Haber et al., 2004; Chen et al., 2007; Padhukasahasram and Rannala, 2013). Therefore, the propensity to resolve DSBs with X–Y gene conversion might result in a similar propensity for ectopic X–Y crossing-over. Interestingly, most of the X-to-Y GCHs so far identified overlap sites in which an illegitimate X–Y crossing-over has been previously described (Vollrath et al., 1992; Schiebel et al., 1997; Trombetta et al., 2014). This situation is similar to that observed by Lange et al. (2009) within ampliconic sequences, where both crossover and non-crossover (gene conversion) pathways are active between Y chromosome palindrome arms (Lange et al., 2009; Trombetta and Cruciani, 2017). A model has been proposed in which Y–Y gene conversion may be useful in protecting Y chromosome against its evolutionary degradation (Charlesworth, 2003; Rozen et al., 2003; Connallon and Clark, 2010; Marais et al., 2010; Betrán et al., 2012; Hallast et al., 2013; Trombetta and Cruciani, 2017), by facilitating the efficient removal of Y-linked deleterious alleles from the population. This model can be difficult to apply to X-to-Y gene conversion. Most theories regarding the evolutionary differentiation of X and Y chromosomes posit that recombination between them will be costly because of functional differences between the X-linked and Y-linked gene copies (Jordan and Charlesworth, 2012; Charlesworth et al., 2014). For those genes where the optimal sequence is the same on the X and Y, gene conversion might be beneficial by aiding the removal of Y-linked deleterious mutations, yet many other genes might not fit this constraint, and for these, gene conversion would be costly, due to the introduction of X-borne variants on the Y-linked genes. However, it could be argued that, before DNA replication, no homologous sequences can be used by the “haploid” MSY to repair DSBs in males. Therefore, X-to-Y gene conversion may represent the *extrema ratio* for the haploid male sequences to maintain their integrity. In this view, we can hypothesize that the observed GCHs may be regions of the MSY characterized by structural instability and that gene conversion is the molecular pathway which repairs these regions. However, the excess of exonic sequences covered by the GCHs (Trombetta et al., 2014), the occurrence of shared active GCHs between humans and chimpanzees (Trombetta et al., 2010, 2014, 2016) and the expression of Y genes in many tissues (Prokop and Deschepper, 2015) would not exclude a role for selection in maintaining a beneficial effect of at least some GCHs.

X-to-Y gene conversion rate estimates range from a minimum mean value of 1.8 × 10^-8^ to a maximum of 1.1 × 10^-6^ conversion/base/generation (Cruciani et al., 2010; Trombetta et al., 2014). The estimates of X-to-Y gene conversion rate are considerably lower than that reported for Y–Y gene conversion (Rozen et al., 2003; Hallast et al., 2013), but similar or even much higher than recent estimates of MSY mutation rates (Francalacci et al., 2013; Mendez et al., 2013; Poznik et al., 2013; Scozzari et al., 2014; Helgason et al., 2015; Trombetta et al., 2015). Thus, although there are not enough GCHs to affect the overall mutation rate of human MSY (Helgason et al., 2015), it is clear that ICGC can be highly effective in increasing the level of diversity at specific hotspots.

## Consequence of Icgc in the Interpretation of the Msy Diversity

Excluding the GCHs, the MSY is mainly composed of recombinationally inert regions, whose genetic diversity is essentially due to the sequential accumulation of new mutations. Over generations, the accumulation of different variants has made it possible to define specific haplogroups of MSY characterized by diagnostic mutations (Underhill and Kivisild, 2007). Due to the low mutation rate of the MSY, the haplogroups can be considered evolutionarily stable entities and can be organized in an unambiguous phylogenetic tree (Scozzari et al., 2014; Karmin et al., 2015; Poznik et al., 2016). The human MSY tree is an essential tool for investigating many issues including forensic science (Jobling et al., 1997), human evolution (Jobling and Tyler-Smith, 2003), and medical genetics (Krausz et al., 2004). The presence of gene conversion potentially raises some questions about the use of SNPs found in GCHs as stable markers in the construction of the phylogenetic tree of the Y chromosome and their use in forensic applications. Indeed, gene conversion can produce phylogenetically incoherent SNPs creating the same derived polymorphism in several branches of the MSY phylogeny, or it can change the derived state of a SNP in its ancestral state (Adams et al., 2006; Trombetta et al., 2010, 2014, 2016; Hallast et al., 2013; Niederstätter et al., 2013). Actually, in recently published Next Generation Sequencing (NGS) studies, where thousands of SNPs are used, different nodes of the phylogeny are often supported by multiple markers, so that phylogenetically inconsistent SNPs created by ICGC can be easily identified and removed. For example, in a recent NGS study (Karmin et al., 2015) several variants identified within the highly polymorphic LTR2 GCH have been identified and discarded due to their phylogenetic inconsistency (**Table 1**).

X-to-Y (or autosome-to-Y) gene conversion does not just increase the nucleotide diversity of the MSY, but also generates a significant excess of clustered SNPs (defined as groups of two or more SNPs occurring in close proximity and on the same branch of the Y phylogeny) on GCHs (Trombetta et al., 2016).

The presence of clustered SNPs casts some doubts on the use of NGS to identify new polymorphisms within GCHs. Since the length of the ICGC tracts is comparable with the length of the reads generated by NGS, it is possible that a “converted” sequence can be confused with the donor sequence and wrongly aligned to the paralogous region. Alternatively, the reads could be discarded by the alignment processes or deep-sequencing-associated bioinformatics analyses may consider clustered SNPs as false positives and discard them. This implies that clustered mutations could be lost in NGS re-sequencing studies and that the impact of gene conversion on the diversity of the MSY (and possibly of the entire human genome) could have been underestimated.

## Author Contributions

BT conceived and wrote the manuscript; ED co-wrote the manuscript and handled the reference section; FC conceived and critically revised the manuscript. All authors read and approved the final manuscript.

## Conflict of Interest Statement

The authors declare that the research was conducted in the absence of any commercial or financial relationships that could be construed as a potential conflict of interest.
